# Supplementary material for: PH domain-mediated autoinhibition and oncogenic activation of Akt
Source: eLife. 2022 Aug 15;11:e80148. doi: 10.7554/eLife.80148 (PMC9417420; doi:10.7554/eLife.80148)
Supplement: Figure 1—source data 1. [file elife-80148-fig1-data1.zip › Figure 1-source data 1_gel image with labelling.pdf]

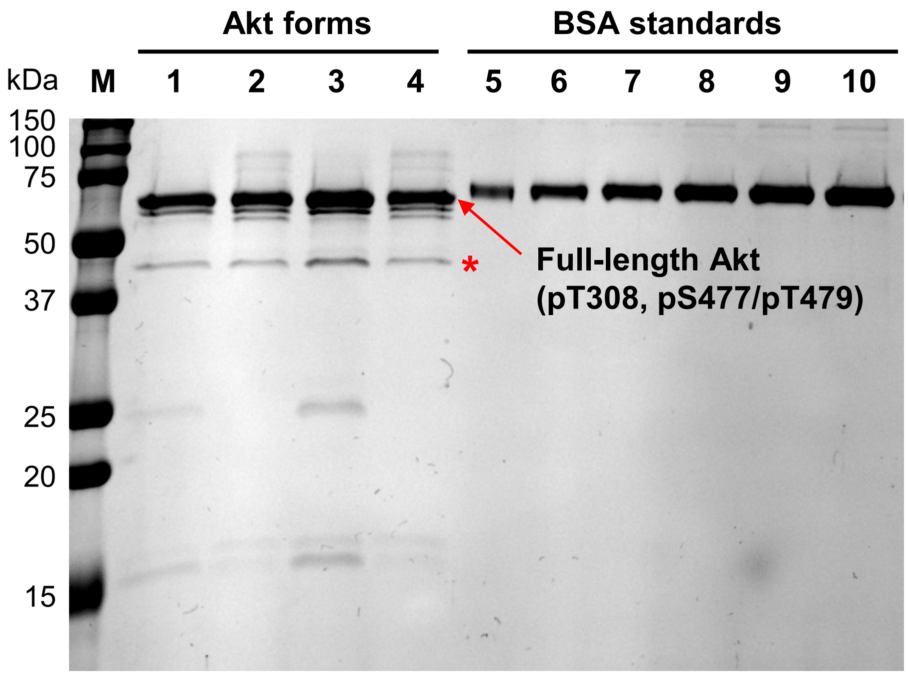

The purity and concentration of each Akt form determined using SDS-PAGE followed by Coomassie staining. lane 1: WT, lane 2: R86A, lane 3: K30A R48A, lane 4: R15A K20A R67A, lane 5–10: BSA standards 0.1, 0.2, 0.3, 0.4, 0.5, 0.6 μg, M: protein markers (kDa). * Mark indicates PreScission Protease added to cleave the N-terminal tags, which does not appear to alter the Akt catalytic activities.
