# Supplementary figures and images for: PH domain-mediated autoinhibition and oncogenic activation of Akt
Source: eLife. 2022 Aug 15;11:e80148. doi: 10.7554/eLife.80148 (PMC9417420; doi:10.7554/eLife.80148)

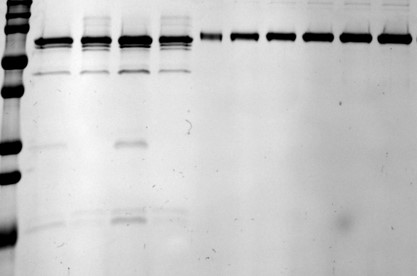

Supplement: Figure 1—source data 1. [file elife-80148-fig1-data1.zip › Figure 1-source data 1_Raw gel image showing the purity and concentration of each Akt mutant.jpg]

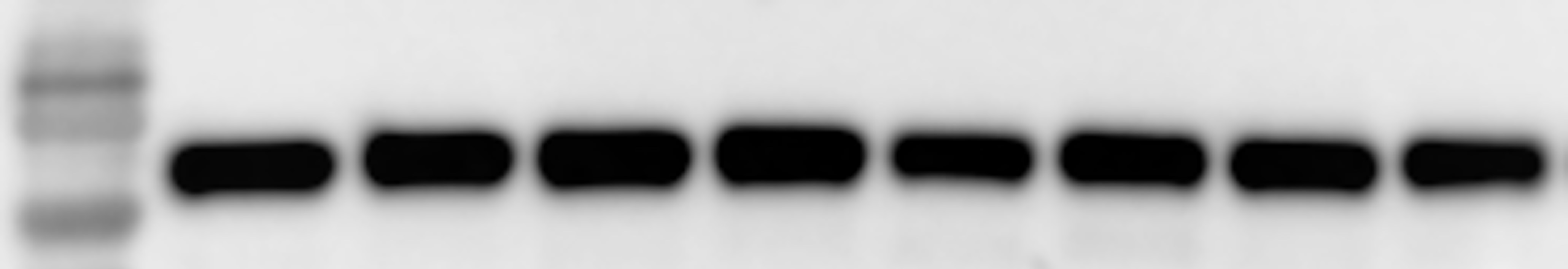

Supplement: Figure 4—figure supplement 2—source data 1. [file elife-80148-fig4-figsupp2-data1.zip › Anti-Akt.jpg]

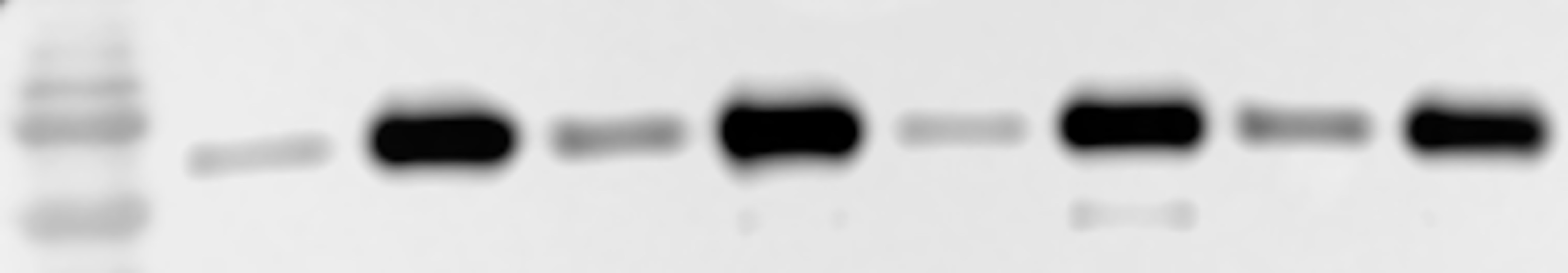

Supplement: Figure 4—figure supplement 2—source data 1. [file elife-80148-fig4-figsupp2-data1.zip › Anti-pS473.jpg]

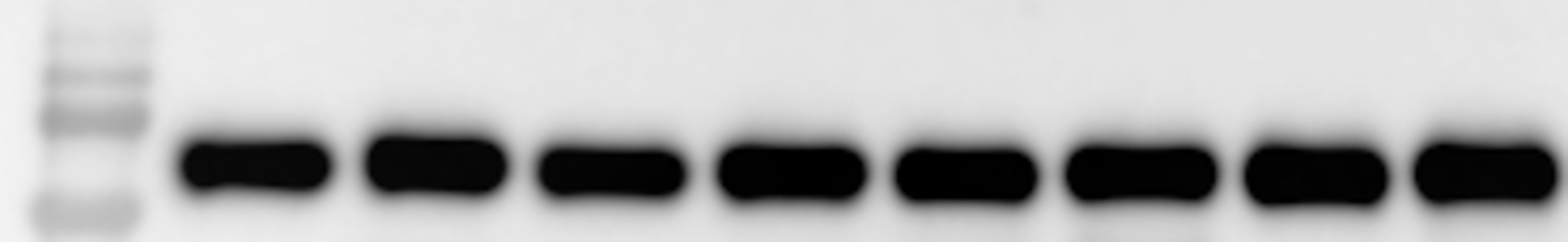

Supplement: Figure 4—figure supplement 2—source data 1. [file elife-80148-fig4-figsupp2-data1.zip › Anti-pT308.jpg]

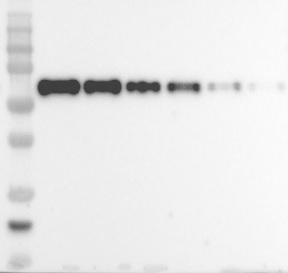

Supplement: Figure 5—source data 2. [file elife-80148-fig5-data2.zip › pT308_E17K-NonP_1.jpg]

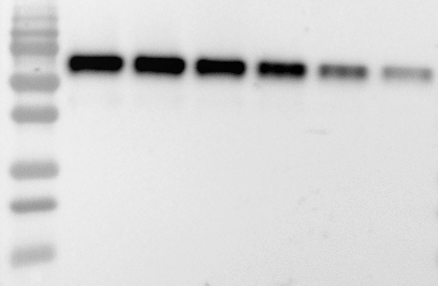

Supplement: Figure 5—source data 2. [file elife-80148-fig5-data2.zip › pT308_E17K-NonP_2.tif]

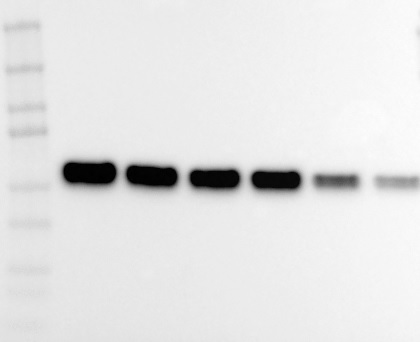

Supplement: Figure 5—source data 2. [file elife-80148-fig5-data2.zip › pT308_E17K-NonP_3.jpg]

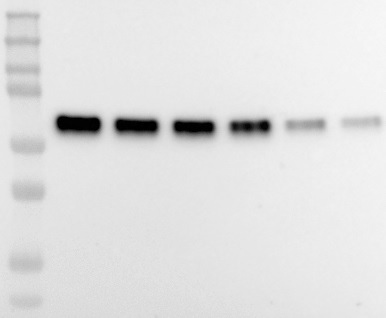

Supplement: Figure 5—source data 2. [file elife-80148-fig5-data2.zip › pT308_E17K-NonP_4.jpg]

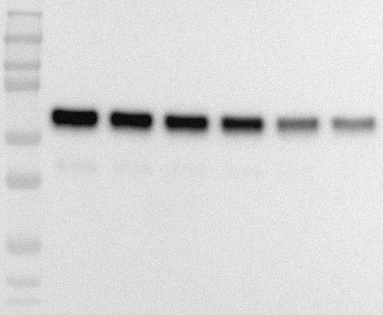

Supplement: Figure 5—source data 2. [file elife-80148-fig5-data2.zip › pT308_E17K-NonP_5.jpg]

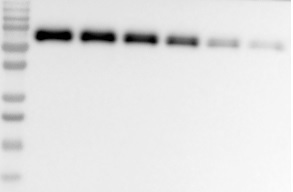

Supplement: Figure 5—source data 2. [file elife-80148-fig5-data2.zip › pT308_R86A-NonP_1.jpg]

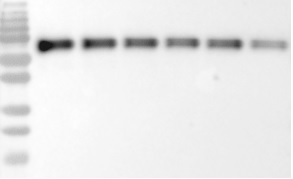

Supplement: Figure 5—source data 2. [file elife-80148-fig5-data2.zip › pT308_R86A-NonP_2.jpg]

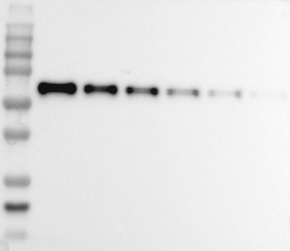

Supplement: Figure 5—source data 2. [file elife-80148-fig5-data2.zip › pT308_R86A-NonP_3.jpg]

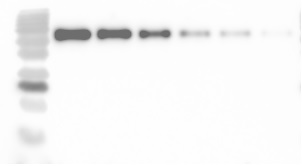

Supplement: Figure 5—source data 2. [file elife-80148-fig5-data2.zip › pT308_WT-NonP_1.jpg]

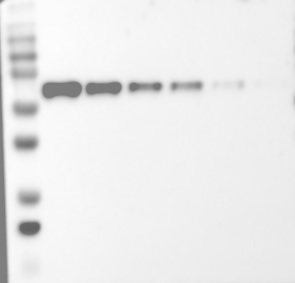

Supplement: Figure 5—source data 2. [file elife-80148-fig5-data2.zip › pT308_WT-NonP_2.jpg]

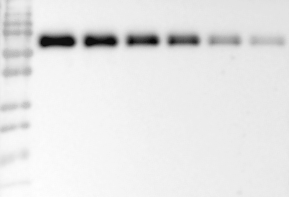

Supplement: Figure 5—source data 2. [file elife-80148-fig5-data2.zip › pT308_WT-NonP_3.tif]

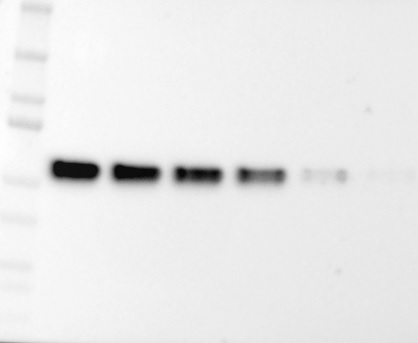

Supplement: Figure 5—source data 2. [file elife-80148-fig5-data2.zip › pT308_WT-NonP_4.jpg]

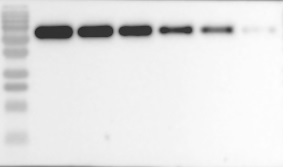

Supplement: Figure 5—source data 2. [file elife-80148-fig5-data2.zip › pT308_Y18A-NonP_1.jpg]

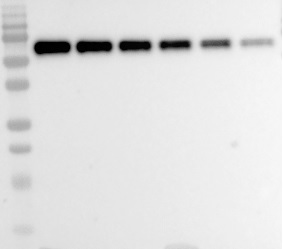

Supplement: Figure 5—source data 2. [file elife-80148-fig5-data2.zip › pT308_Y18A-NonP_2.jpg]

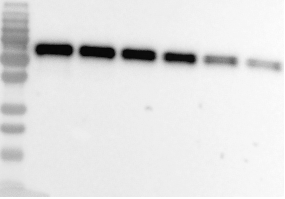

Supplement: Figure 5—source data 2. [file elife-80148-fig5-data2.zip › pT308_Y18A-NonP_3.tif]

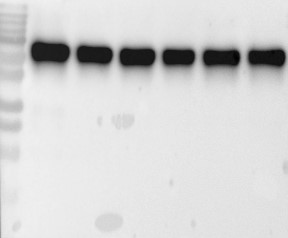

Supplement: Figure 5—source data 2. [file elife-80148-fig5-data2.zip › Total Akt_E17K-NonP.jpg]

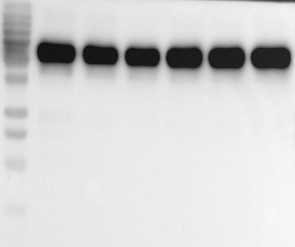

Supplement: Figure 5—source data 2. [file elife-80148-fig5-data2.zip › Total Akt_R86A-NonP.jpg]

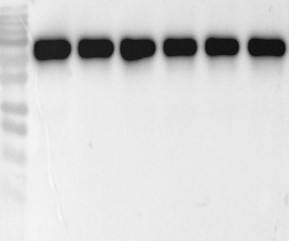

Supplement: Figure 5—source data 2. [file elife-80148-fig5-data2.zip › Total Akt_WT-NonP.jpg]

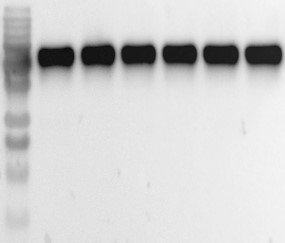

Supplement: Figure 5—source data 2. [file elife-80148-fig5-data2.zip › Total Akt_Y18A-NonP.jpg]

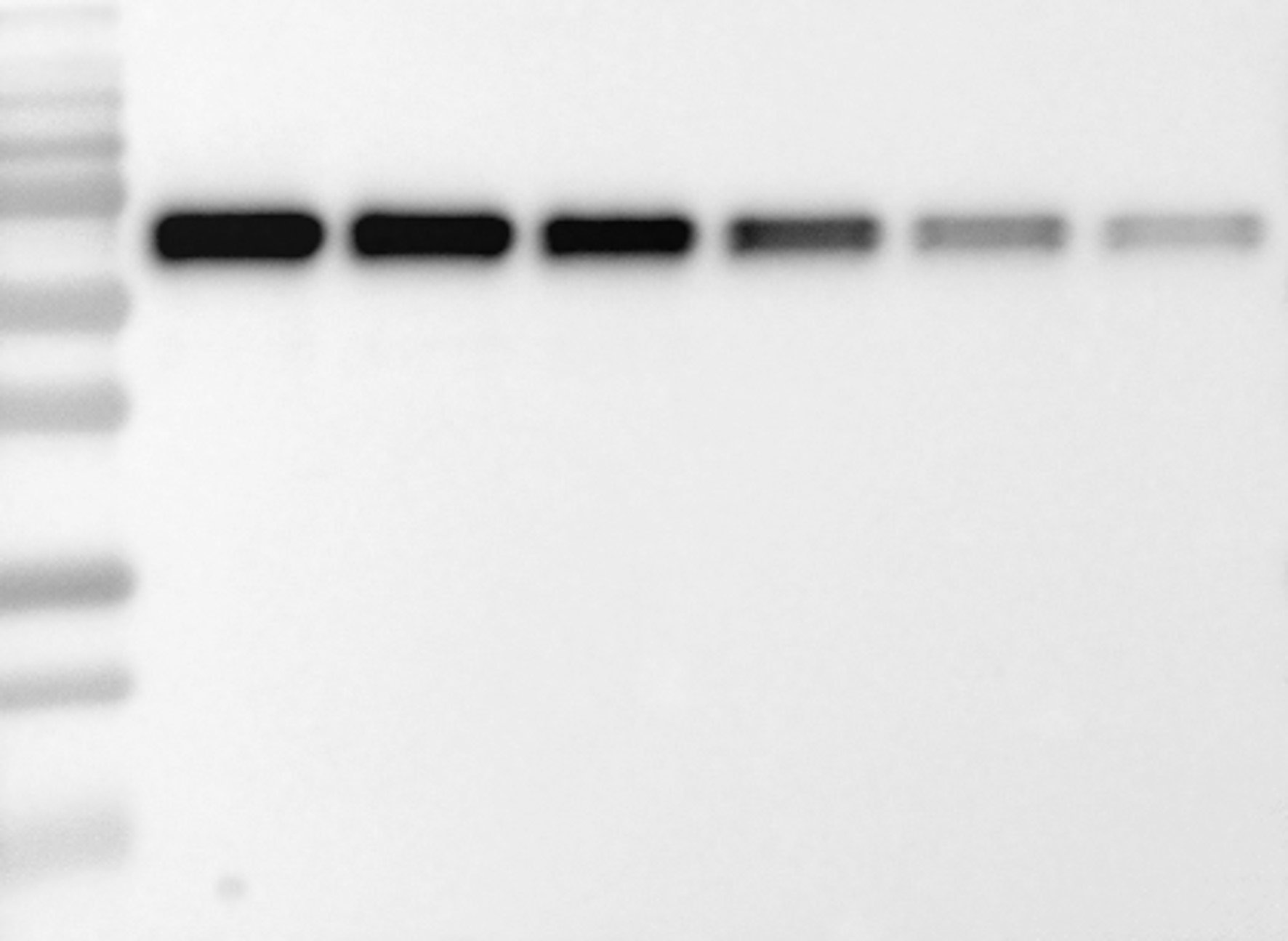

Supplement: Figure 5—figure supplement 1—source data 2. [file elife-80148-fig5-figsupp1-data2.zip › pS473_E17K.jpg]

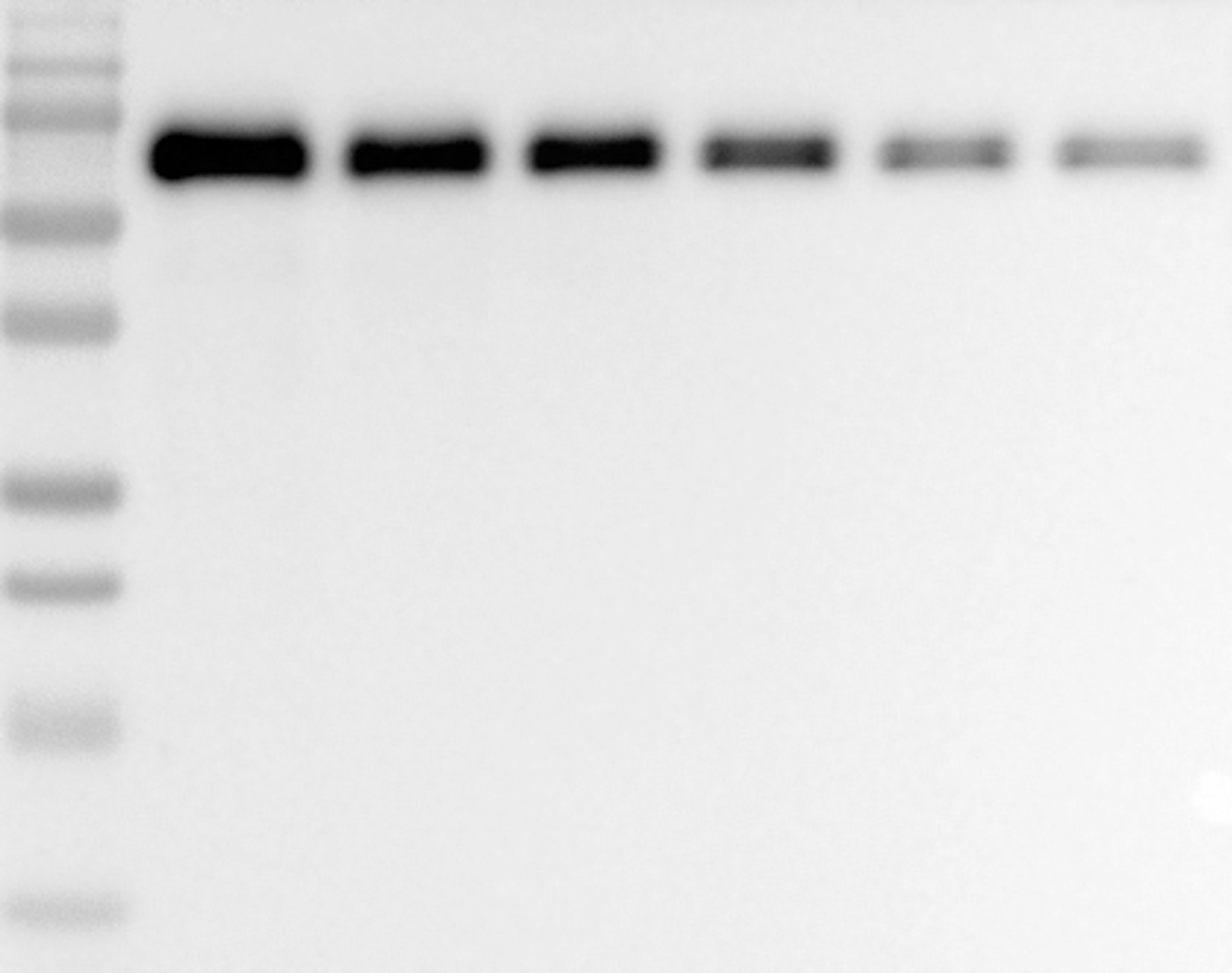

Supplement: Figure 5—figure supplement 1—source data 2. [file elife-80148-fig5-figsupp1-data2.zip › pS473_R86A.jpg]

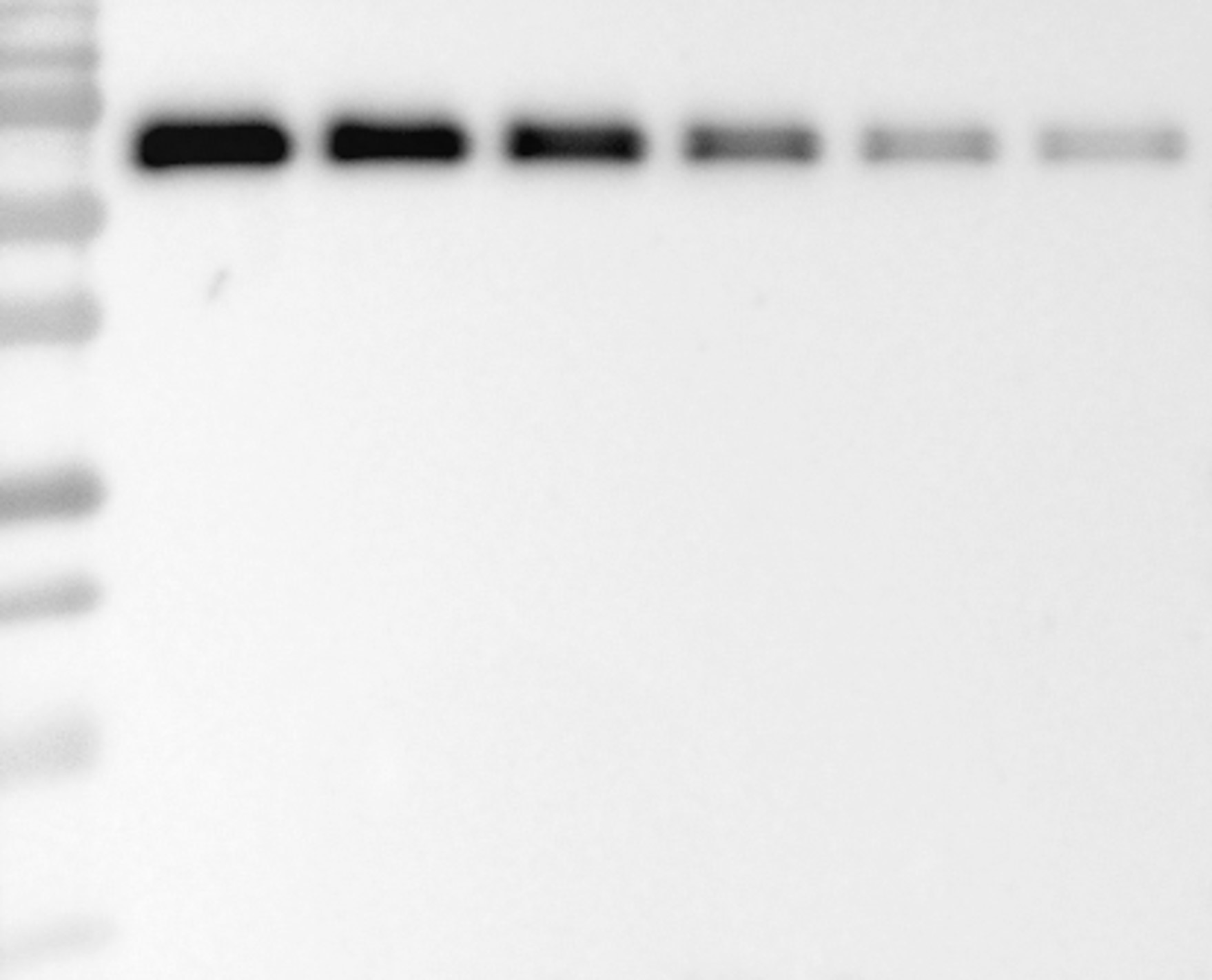

Supplement: Figure 5—figure supplement 1—source data 2. [file elife-80148-fig5-figsupp1-data2.zip › pS473_WT.jpg]

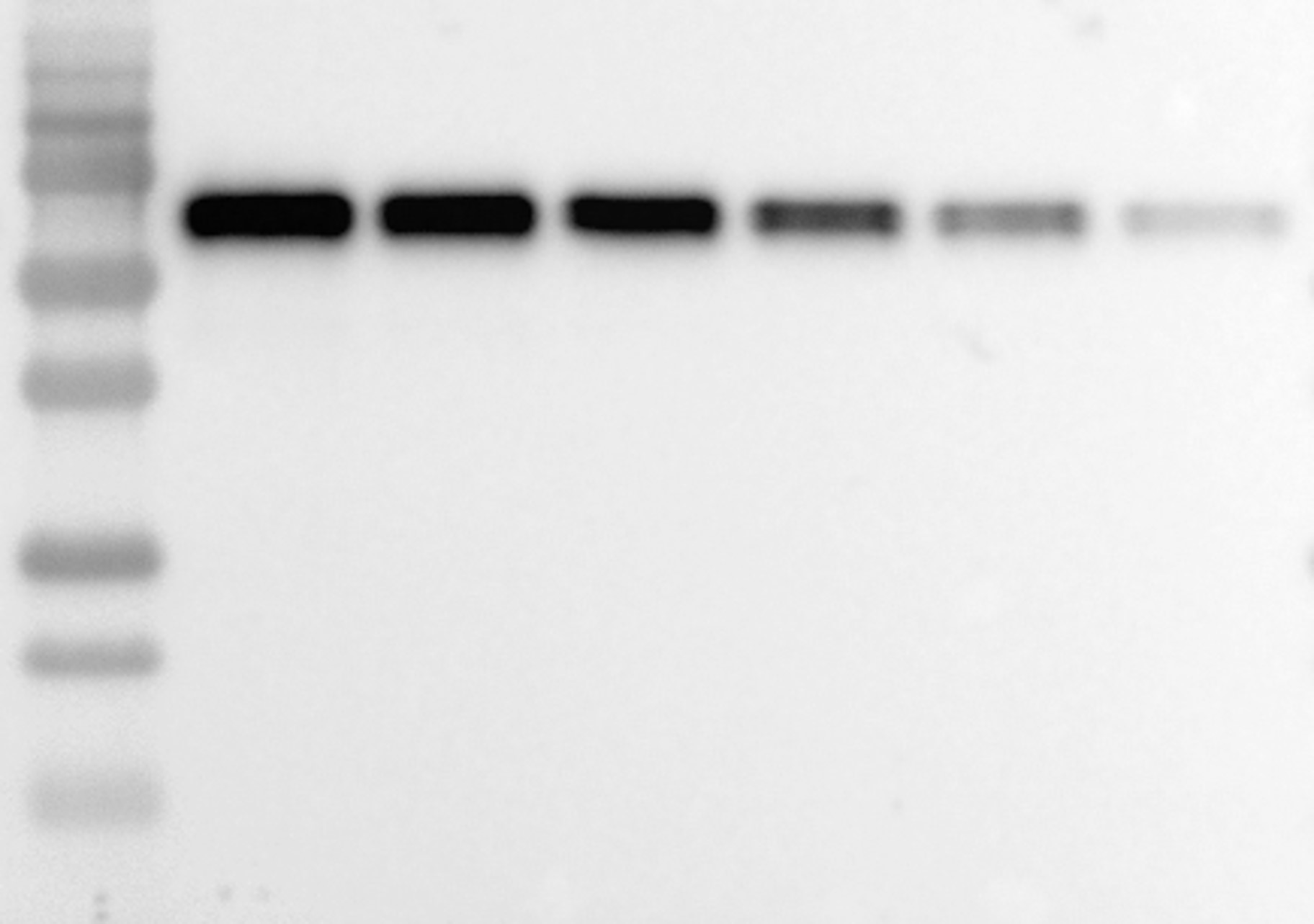

Supplement: Figure 5—figure supplement 1—source data 2. [file elife-80148-fig5-figsupp1-data2.zip › pS473_Y18A.jpg]

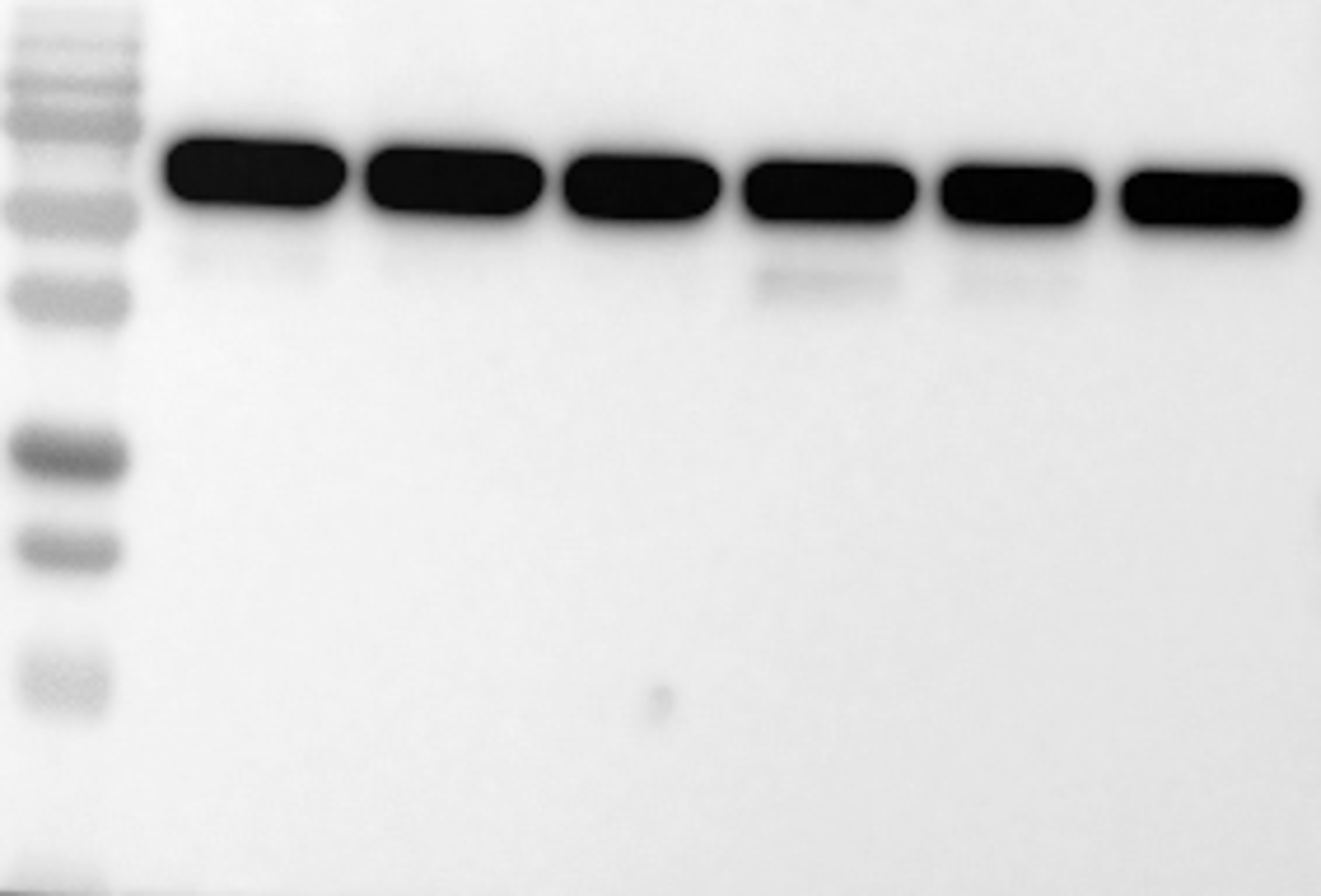

Supplement: Figure 5—figure supplement 1—source data 2. [file elife-80148-fig5-figsupp1-data2.zip › pT308_E17K.jpg]

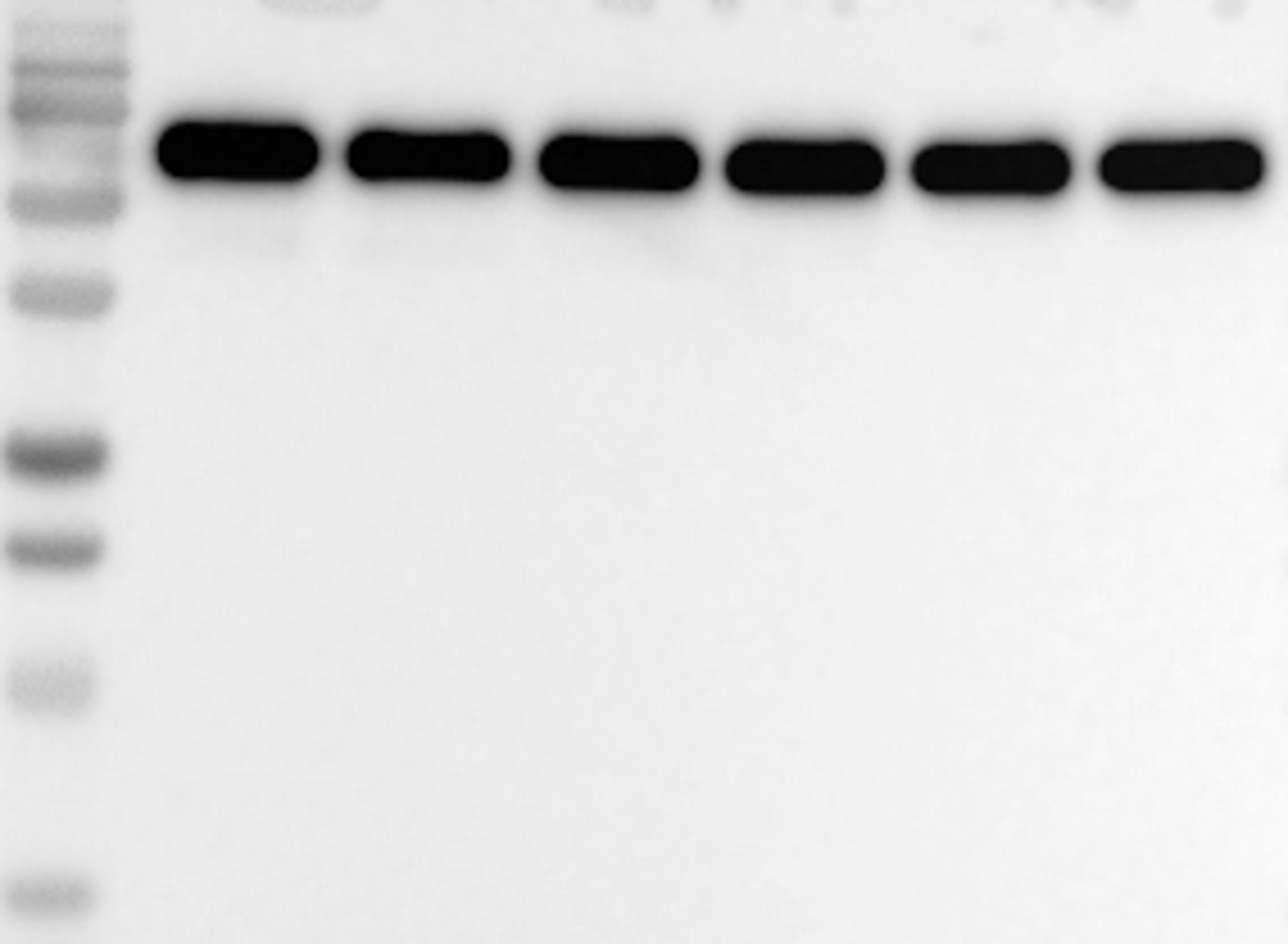

Supplement: Figure 5—figure supplement 1—source data 2. [file elife-80148-fig5-figsupp1-data2.zip › pT308_R86A.jpg]

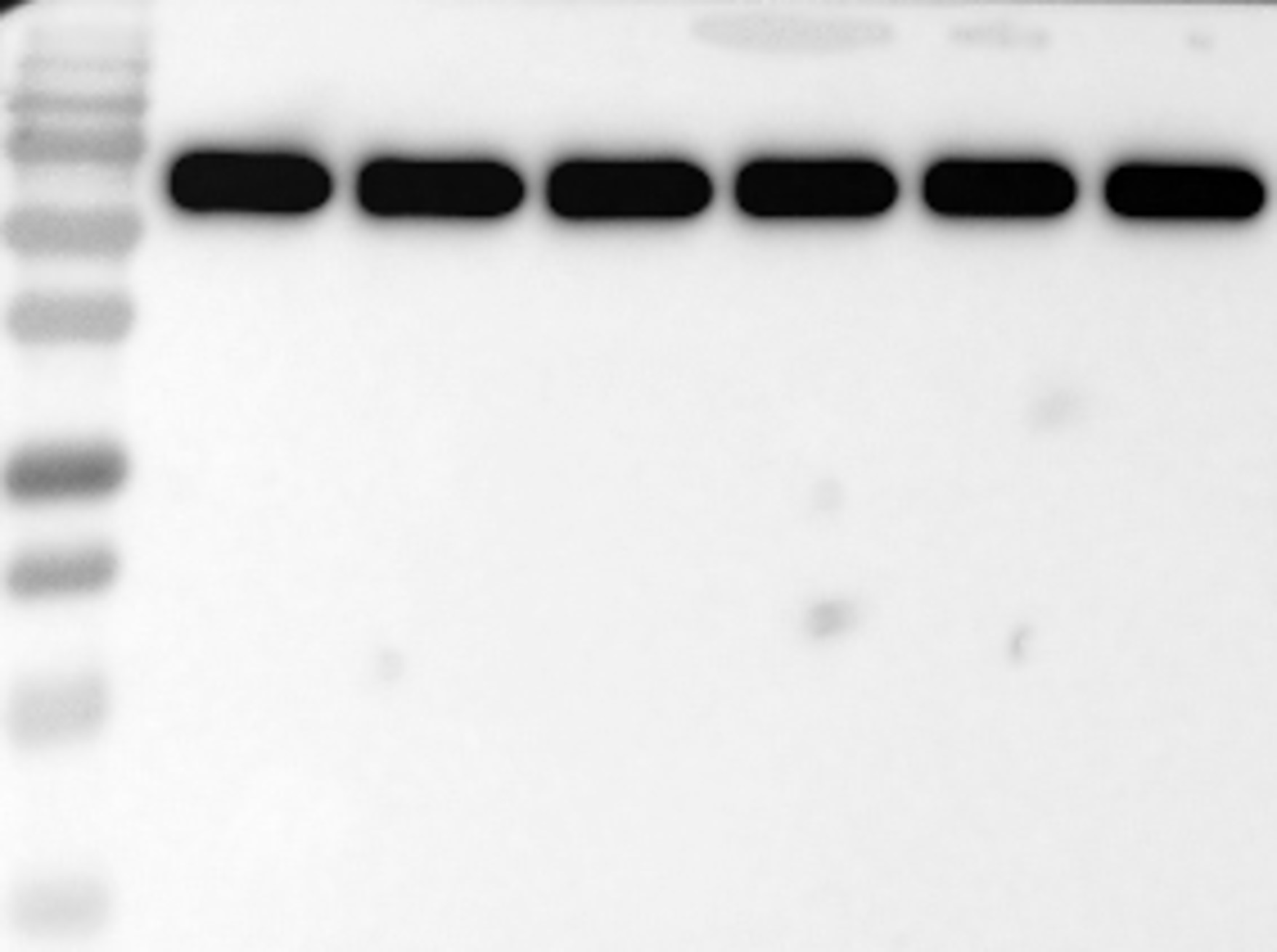

Supplement: Figure 5—figure supplement 1—source data 2. [file elife-80148-fig5-figsupp1-data2.zip › pT308_WT.jpg]

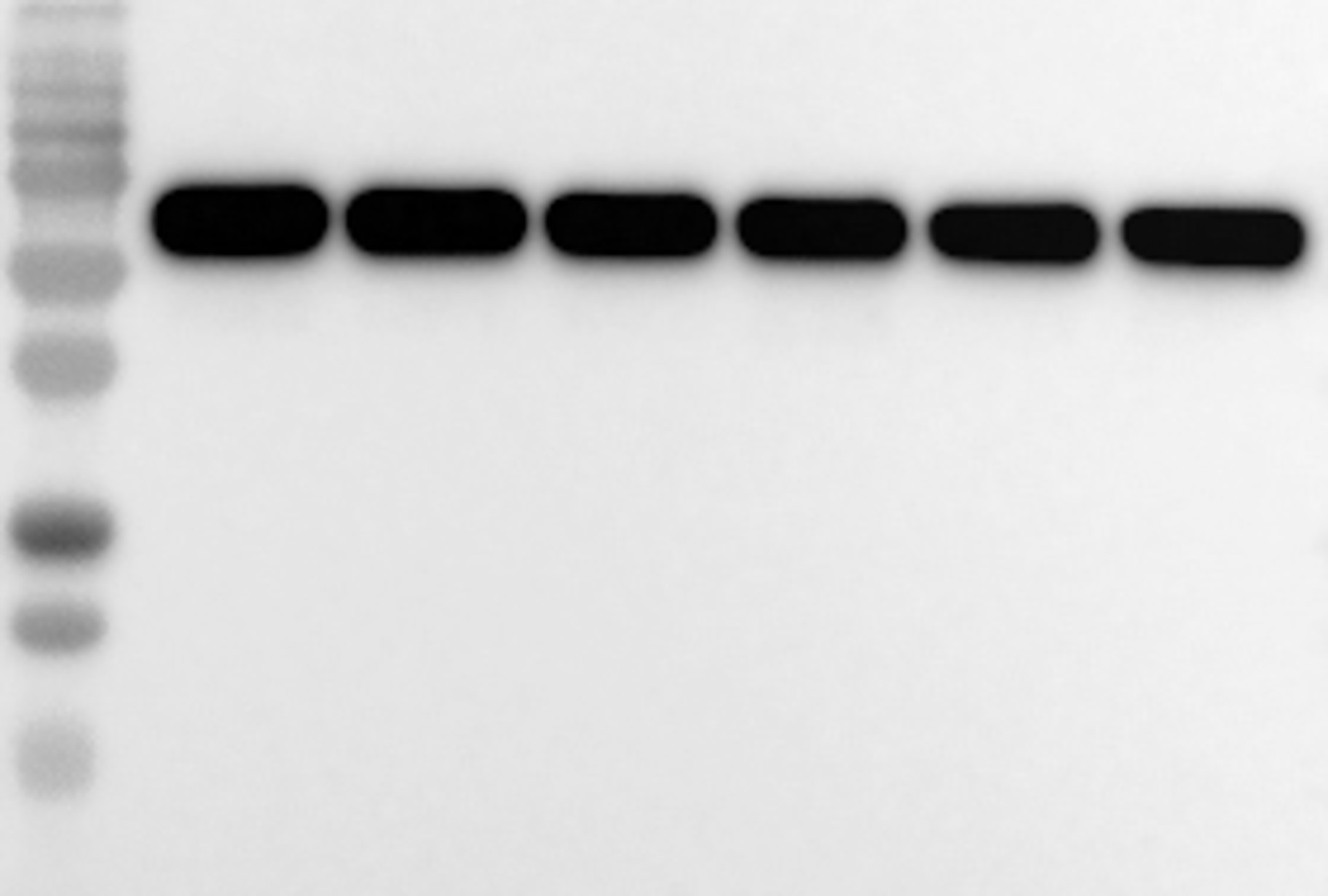

Supplement: Figure 5—figure supplement 1—source data 2. [file elife-80148-fig5-figsupp1-data2.zip › pT308_Y18A.jpg]

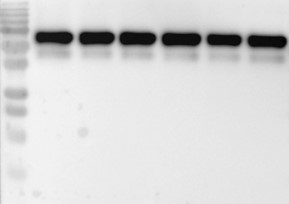

Supplement: Figure 5—figure supplement 1—source data 2. [file elife-80148-fig5-figsupp1-data2.zip › Total Akt_E17K.jpg]

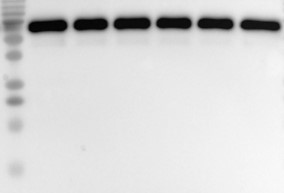

Supplement: Figure 5—figure supplement 1—source data 2. [file elife-80148-fig5-figsupp1-data2.zip › Total Akt_R86A.jpg]

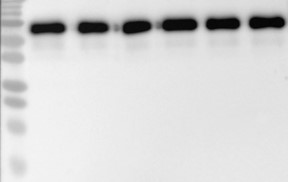

Supplement: Figure 5—figure supplement 1—source data 2. [file elife-80148-fig5-figsupp1-data2.zip › Total Akt_WT.jpg]

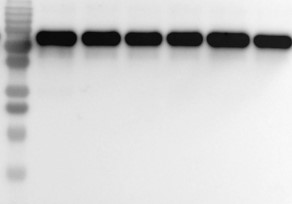

Supplement: Figure 5—figure supplement 1—source data 2. [file elife-80148-fig5-figsupp1-data2.zip › Total Akt_Y18A.jpg]

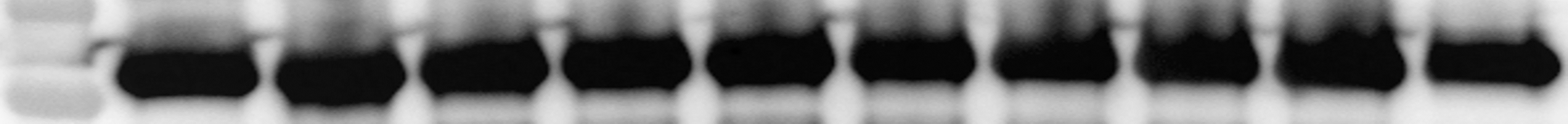

Supplement: Figure 6—figure supplement 3—source data 1. [file elife-80148-fig6-figsupp3-data1.zip › Anti-Akt.png]

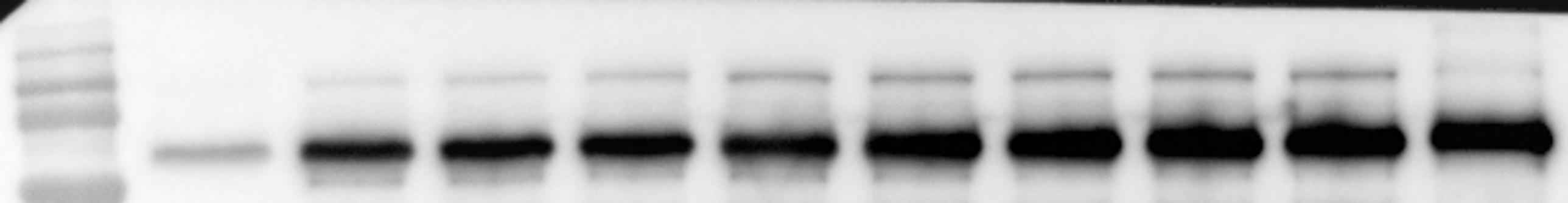

Supplement: Figure 6—figure supplement 3—source data 1. [file elife-80148-fig6-figsupp3-data1.zip › Anti-pT308(CST #13038S).jpg]

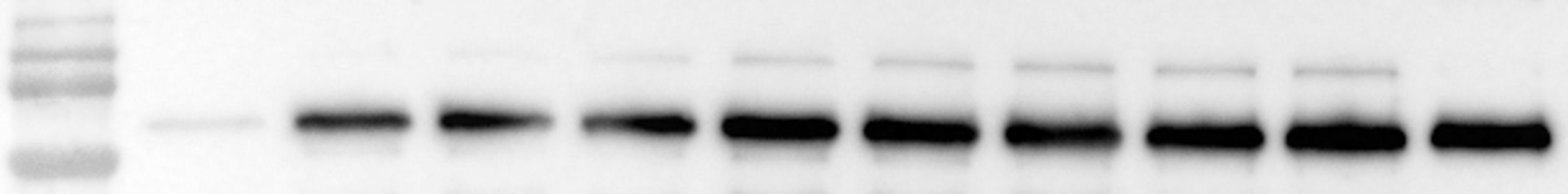

Supplement: Figure 6—figure supplement 3—source data 1. [file elife-80148-fig6-figsupp3-data1.zip › Anti-pT308(CST #9275S).jpg]

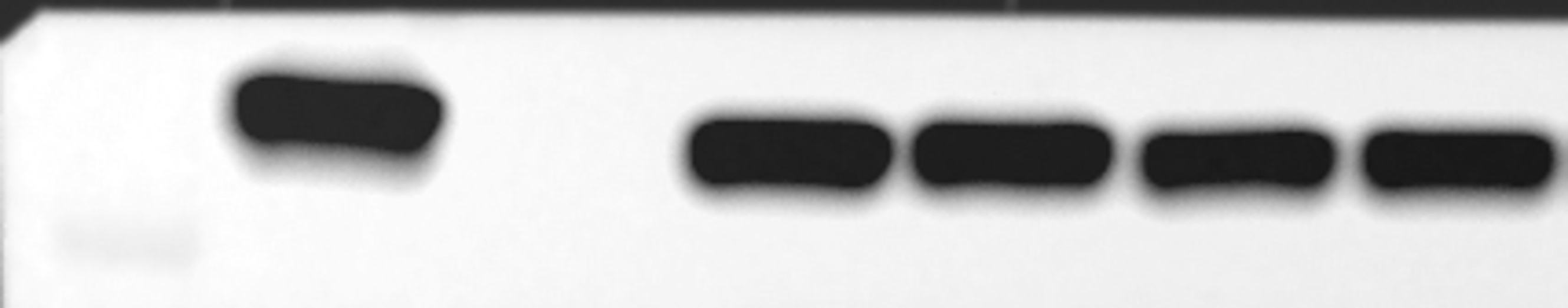

Supplement: Figure 7—source data 1. [file elife-80148-fig7-data1.zip › Akt_1.jpg]

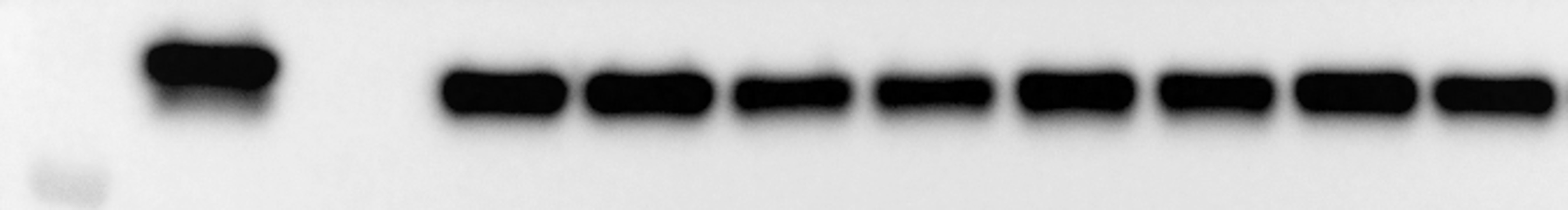

Supplement: Figure 7—source data 1. [file elife-80148-fig7-data1.zip › Akt_2.jpg]

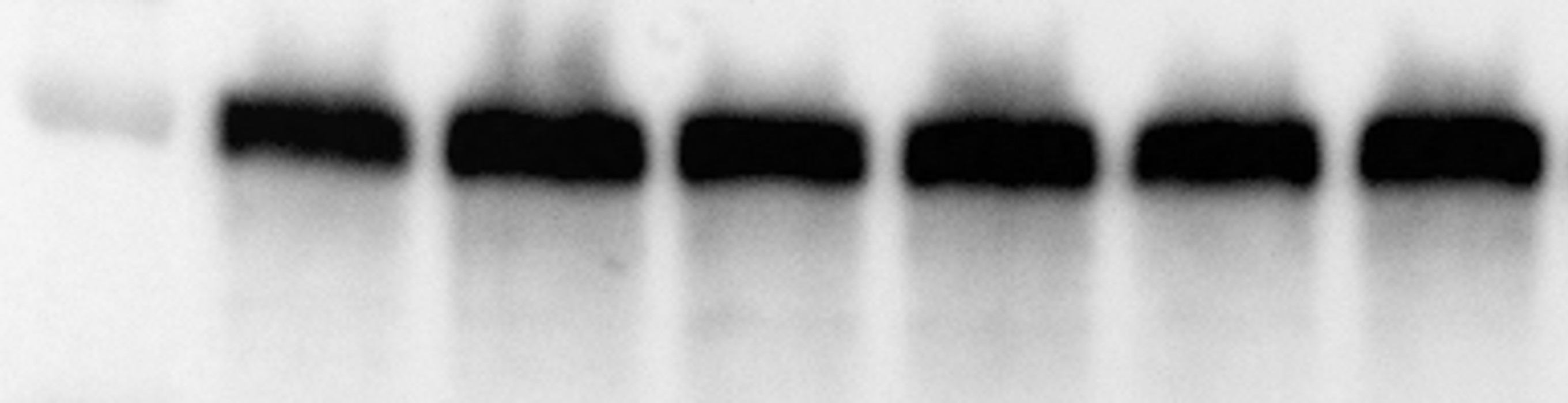

Supplement: Figure 7—source data 1. [file elife-80148-fig7-data1.zip › Foxo1_1.jpg]

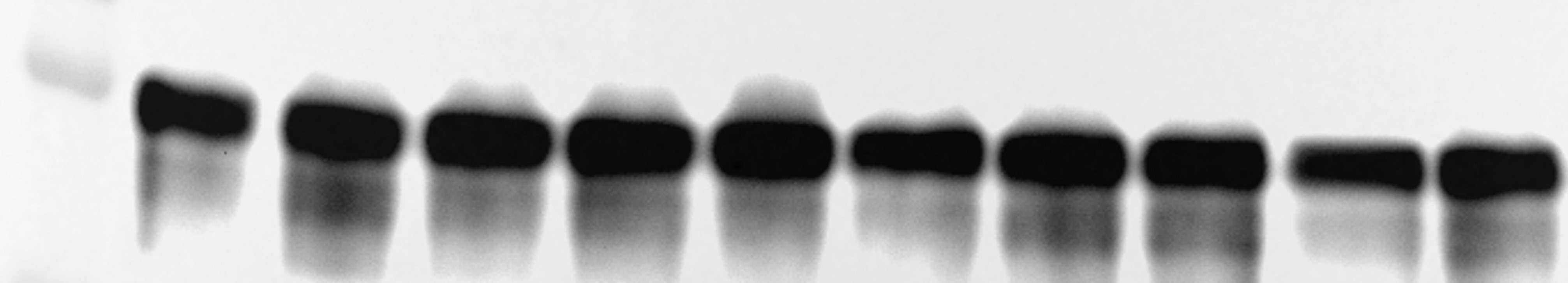

Supplement: Figure 7—source data 1. [file elife-80148-fig7-data1.zip › Foxo1_2.jpg]

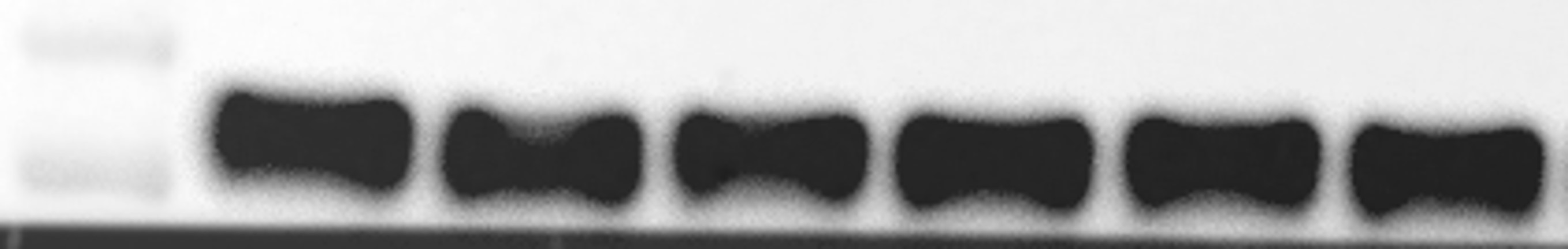

Supplement: Figure 7—source data 1. [file elife-80148-fig7-data1.zip › Foxo3a_1.jpg]

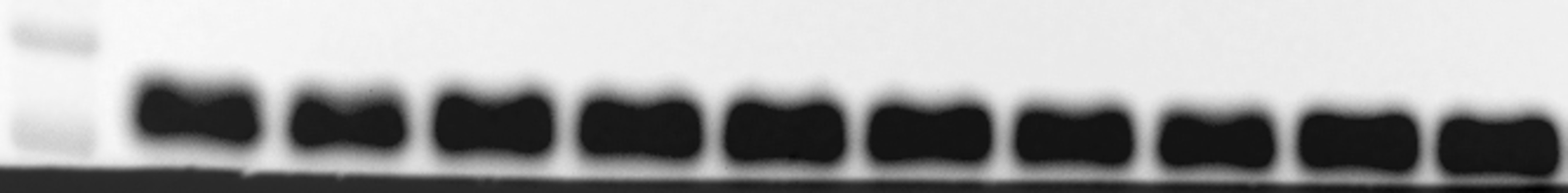

Supplement: Figure 7—source data 1. [file elife-80148-fig7-data1.zip › Foxo3a_2.jpg]

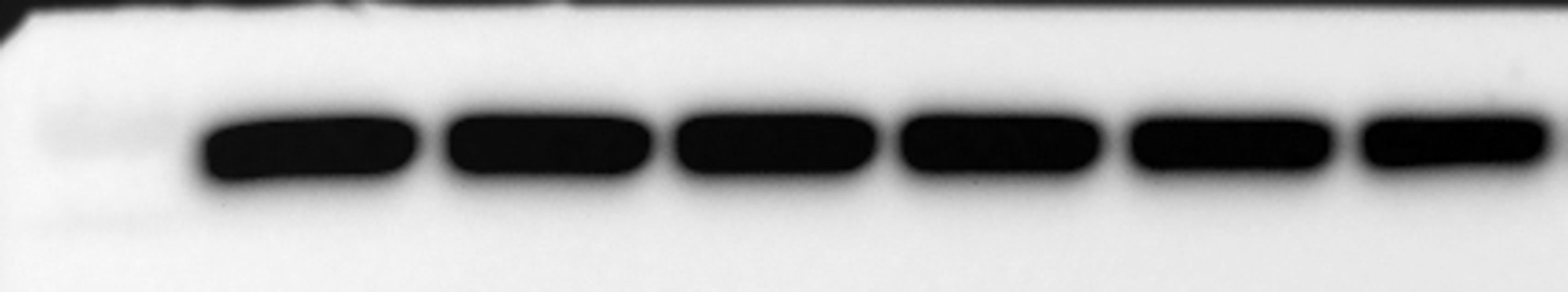

Supplement: Figure 7—source data 1. [file elife-80148-fig7-data1.zip › GAPDH_1.jpg]

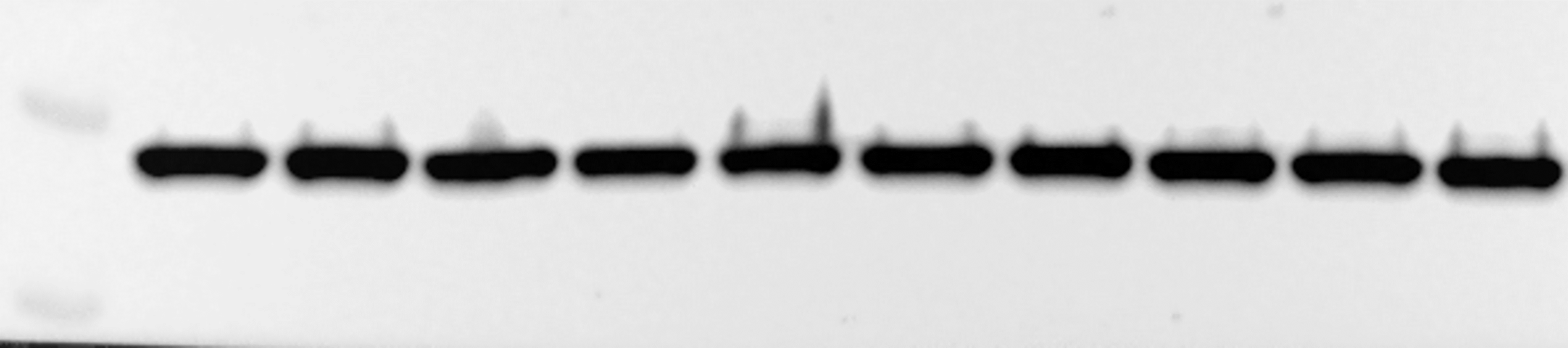

Supplement: Figure 7—source data 1. [file elife-80148-fig7-data1.zip › GAPDH_2.png]

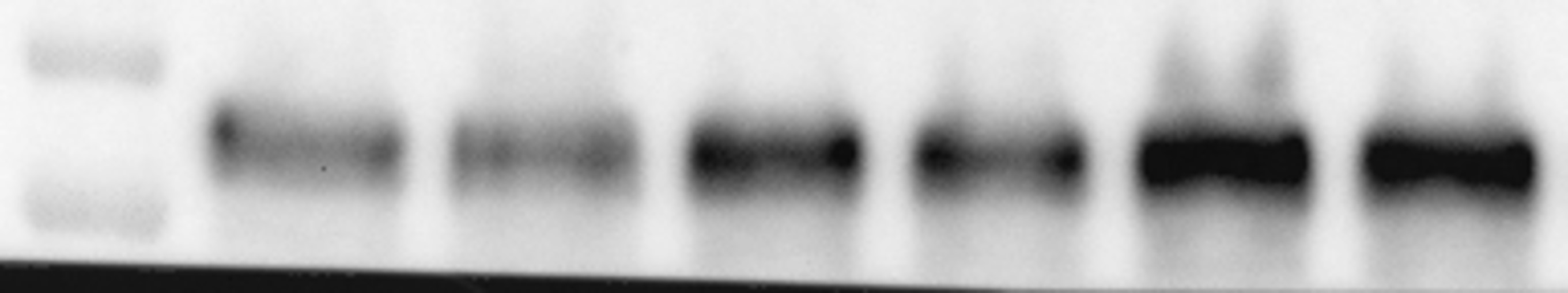

Supplement: Figure 7—source data 1. [file elife-80148-fig7-data1.zip › p-Foxo1_3a_1.jpg]

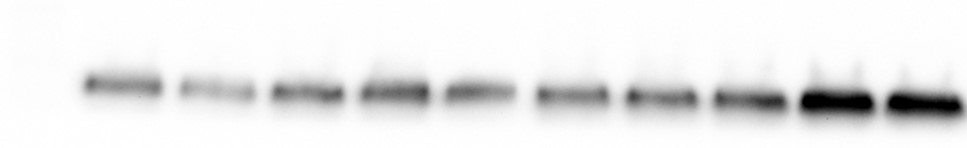

Supplement: Figure 7—source data 1. [file elife-80148-fig7-data1.zip › p-Foxo1_3a_2.jpg]

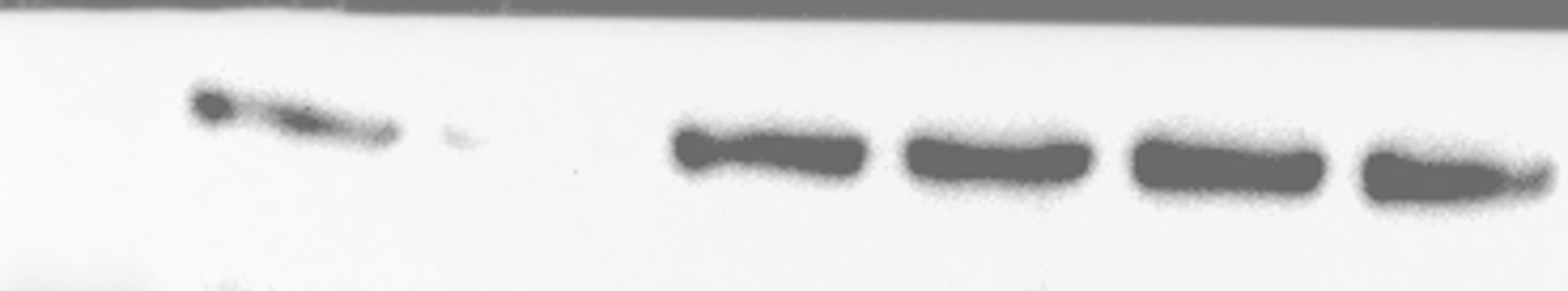

Supplement: Figure 7—source data 1. [file elife-80148-fig7-data1.zip › pT308(Akt)_1.jpg]

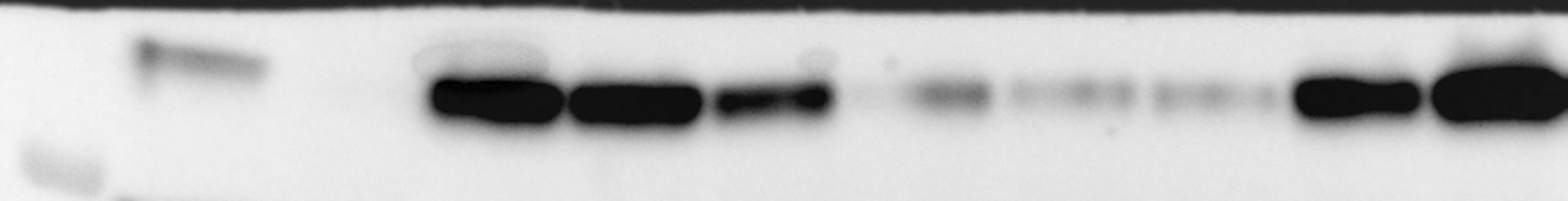

Supplement: Figure 7—source data 1. [file elife-80148-fig7-data1.zip › pT308(Akt)_2.jpg]
