# Supplementary material for: PH domain-mediated autoinhibition and oncogenic activation of Akt
Source: eLife. 2022 Aug 15;11:e80148. doi: 10.7554/eLife.80148 (PMC9417420; doi:10.7554/eLife.80148)

Dephosphorylation by PP2A on pT308 of WT Akt

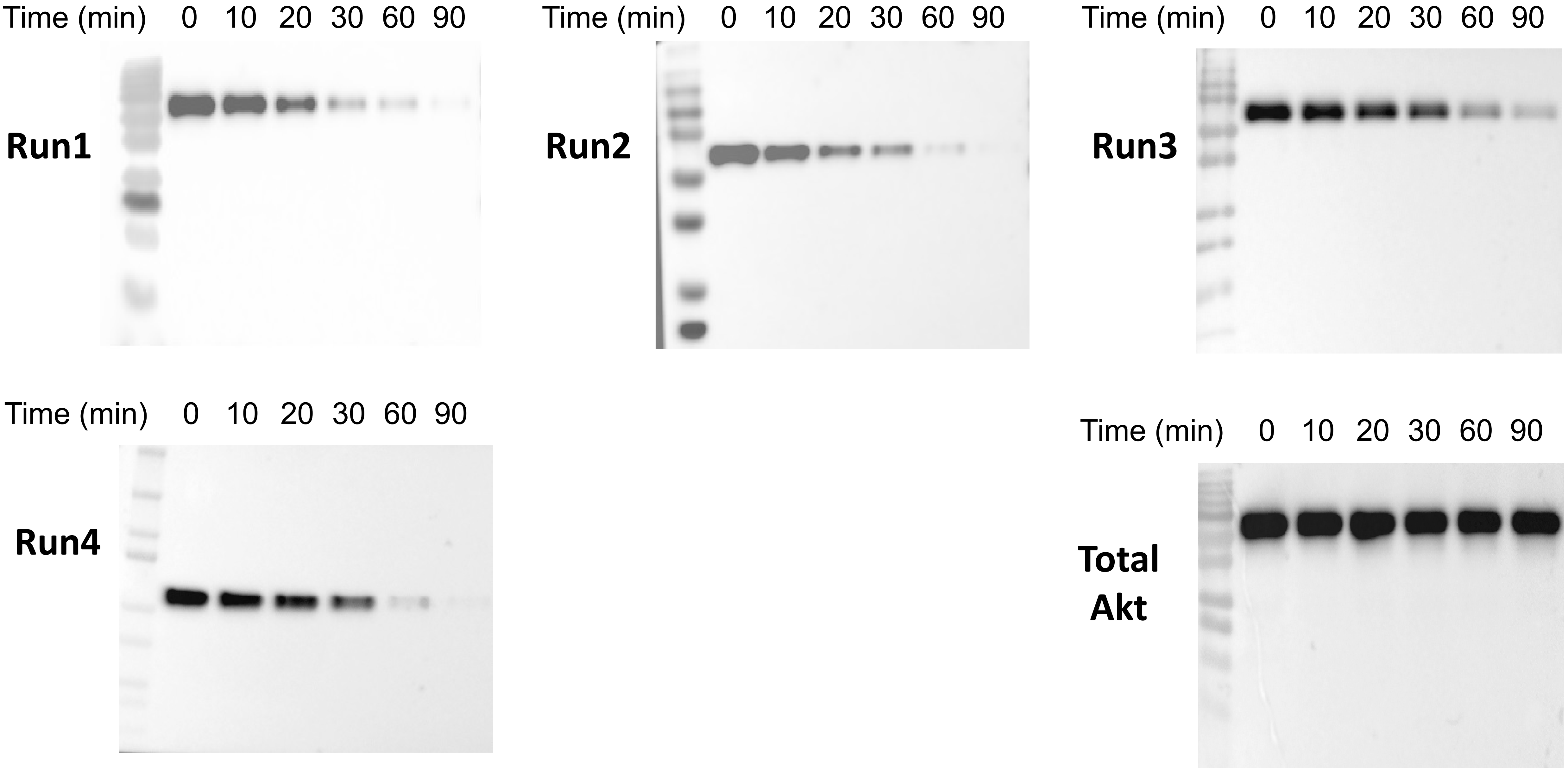

Dephosphorylation by PP2A on pT308 of R86A Akt

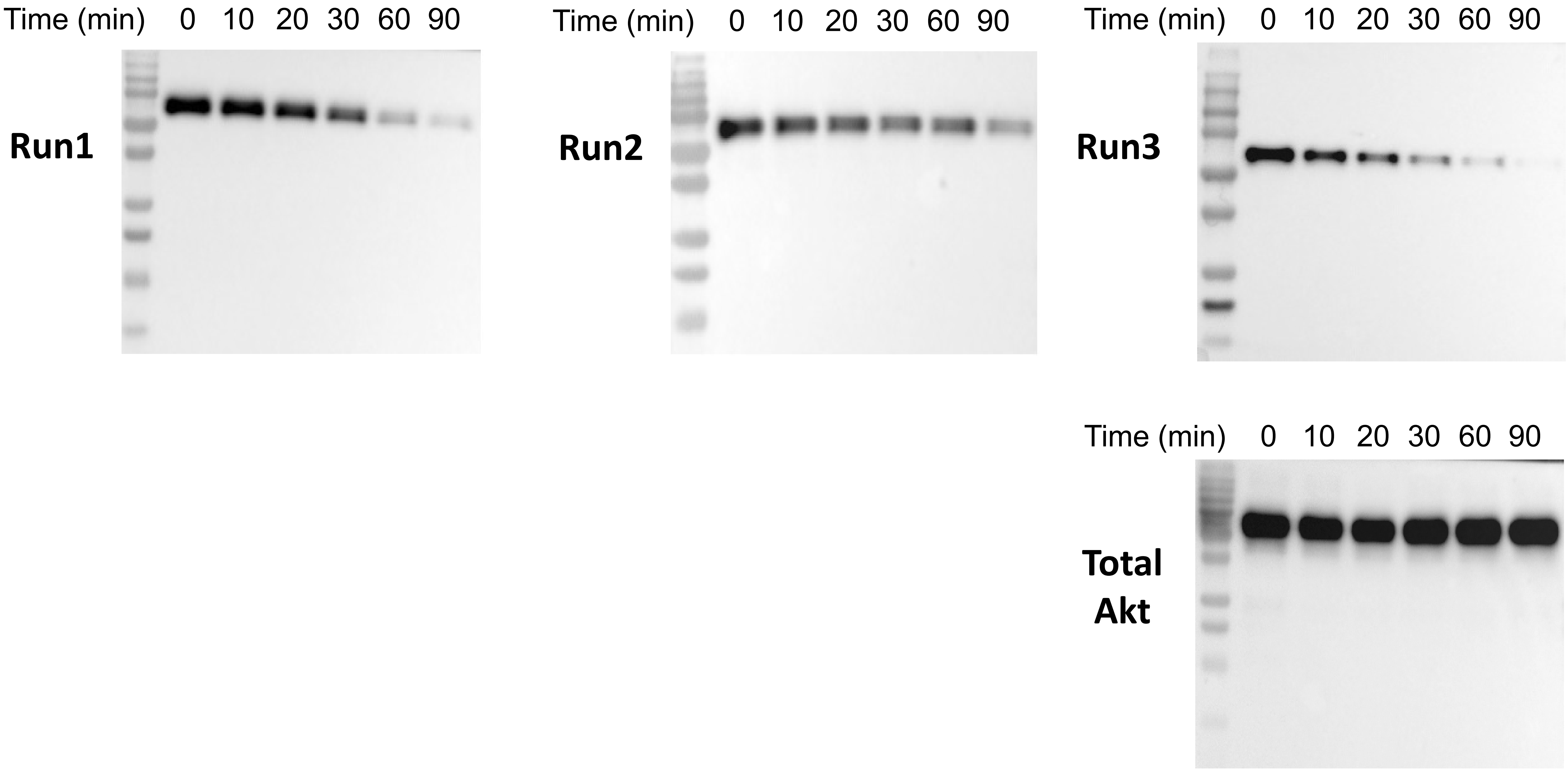

Dephosphorylation by PP2A on pT308 of E17K Akt

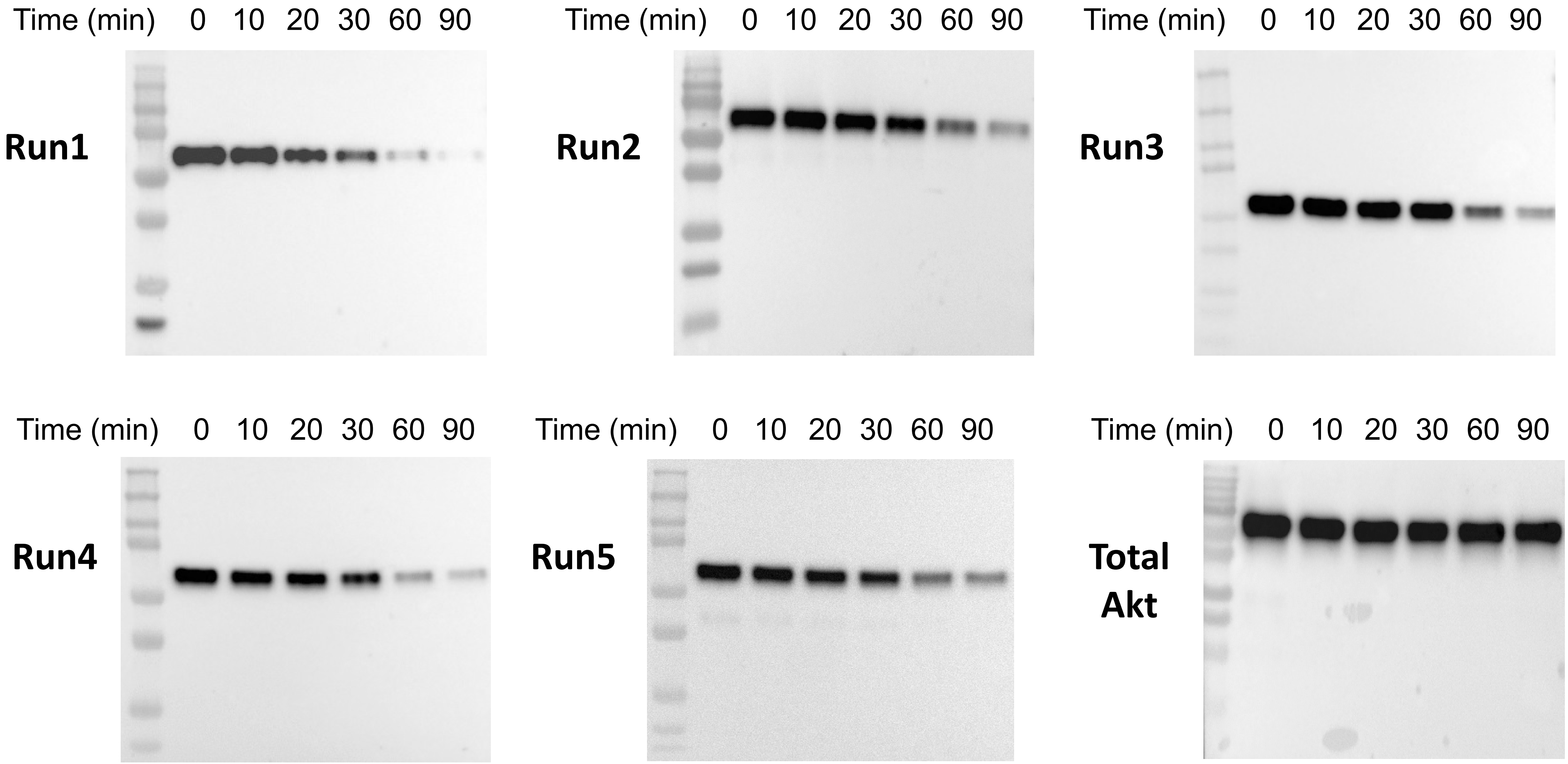

Dephosphorylation by PP2A on pT308 of Y18A Akt

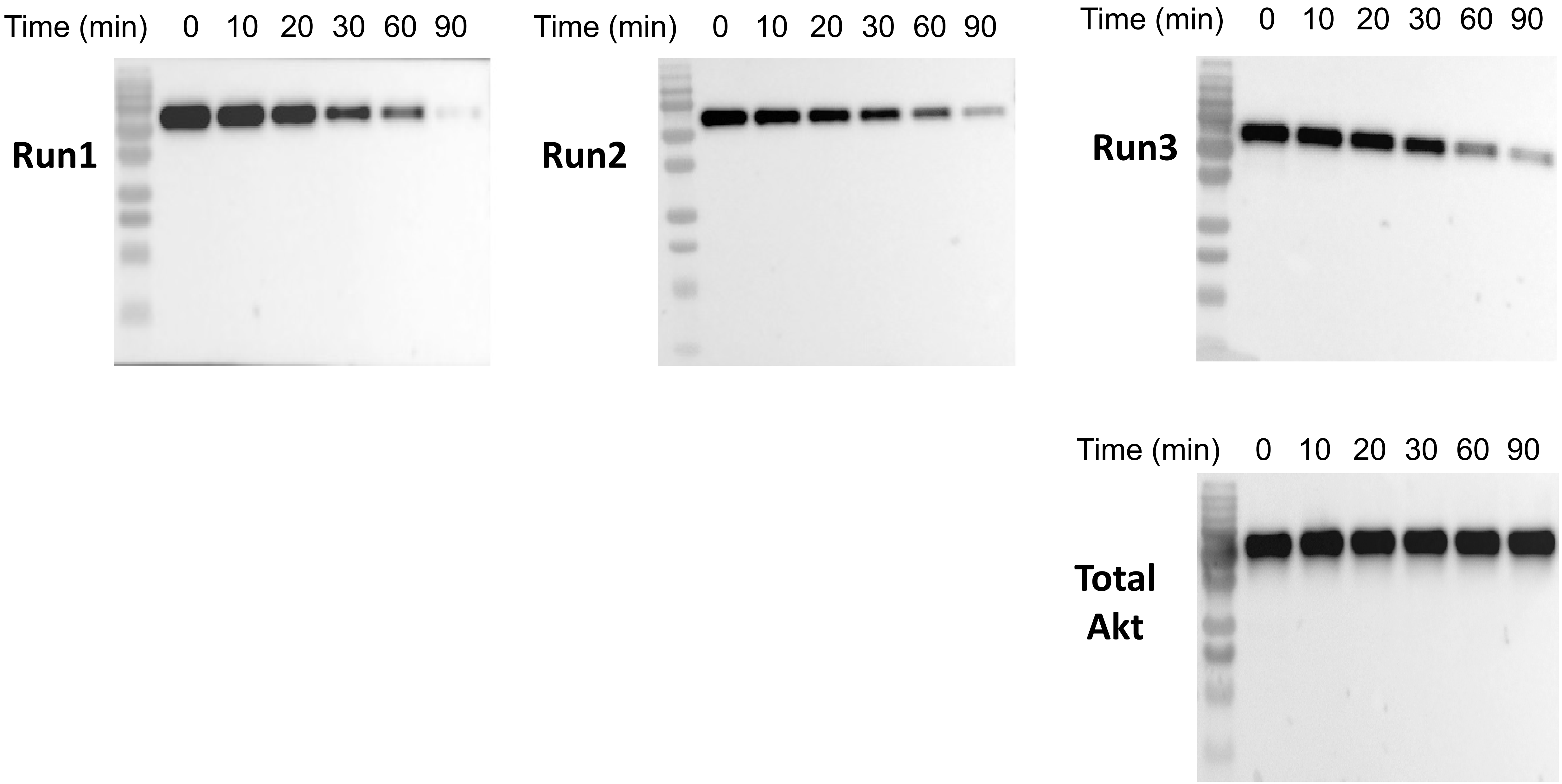

Supplement: Figure 5—source data 2. [file elife-80148-fig5-data2.zip › Figure_5-source_data_Dephosphorylation_assays_by_PP2A_(pT308_WT_R86A_E17K_Y18A).pdf]
