# Supplementary material for: PH domain-mediated autoinhibition and oncogenic activation of Akt
Source: eLife. 2022 Aug 15;11:e80148. doi: 10.7554/eLife.80148 (PMC9417420; doi:10.7554/eLife.80148)

Dephosphorylation by Alkaline PPase on either pT308 or pS473 of WT Akt

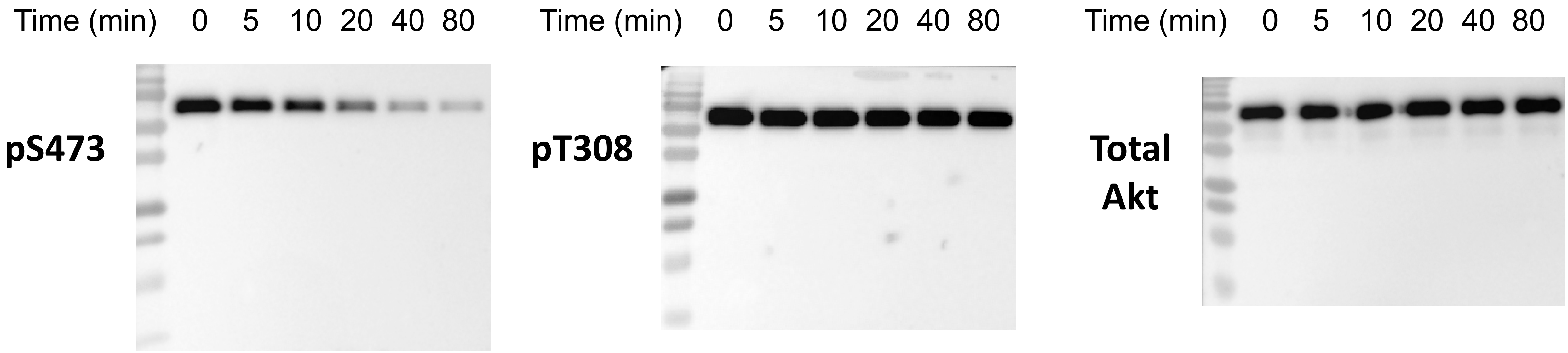

Dephosphorylation by Alkaline PPase on either pT308 or pS473 of R86A Akt

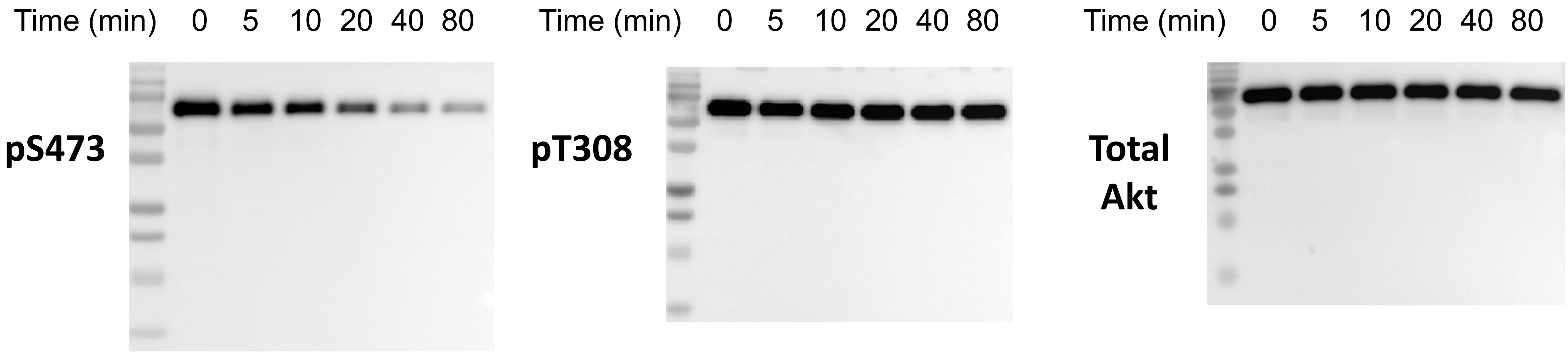

Dephosphorylation by Alkaline PPase on either pT308 or pS473 of E17K Akt

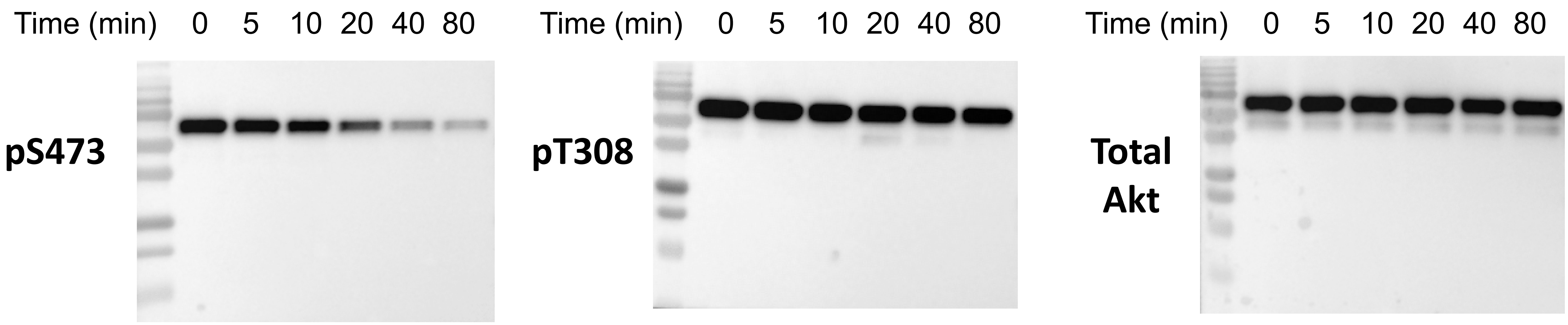

Dephosphorylation by Alkaline PPase on either pT308 or pS473 of Y18A Akt

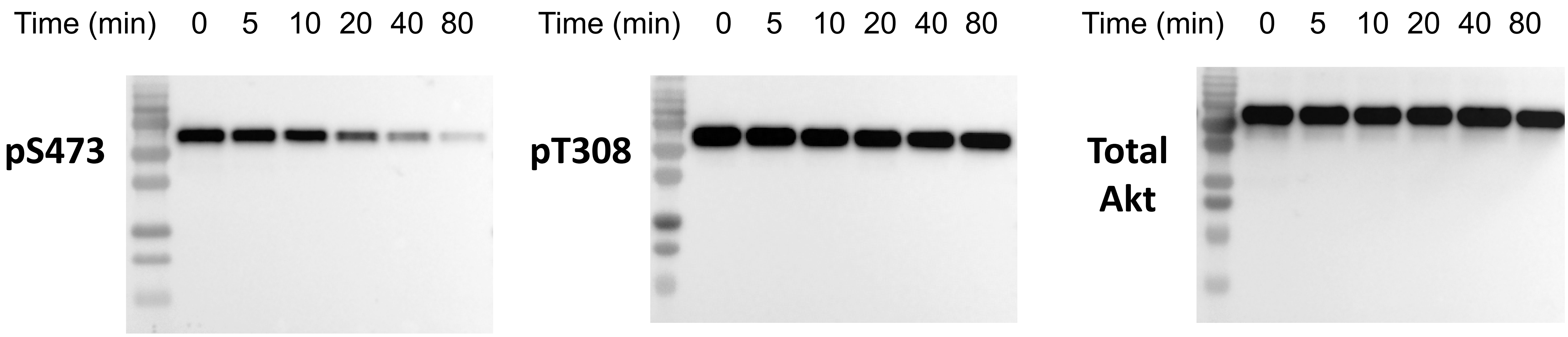

Supplement: Figure 5—figure supplement 1—source data 2. [file elife-80148-fig5-figsupp1-data2.zip › Dephosphorylation by Alkaline PPase (pT308_pS473_WT_R86A_E17K_Y18A).pdf]
