# Supplementary material for: PH domain-mediated autoinhibition and oncogenic activation of Akt
Source: eLife. 2022 Aug 15;11:e80148. doi: 10.7554/eLife.80148 (PMC9417420; doi:10.7554/eLife.80148)
Supplement: Figure 6—figure supplement 3—source data 1. [file elife-80148-fig6-figsupp3-data1.zip › Figure 6-figure supplement 3-source data 1_Western blots with labeling.pdf]

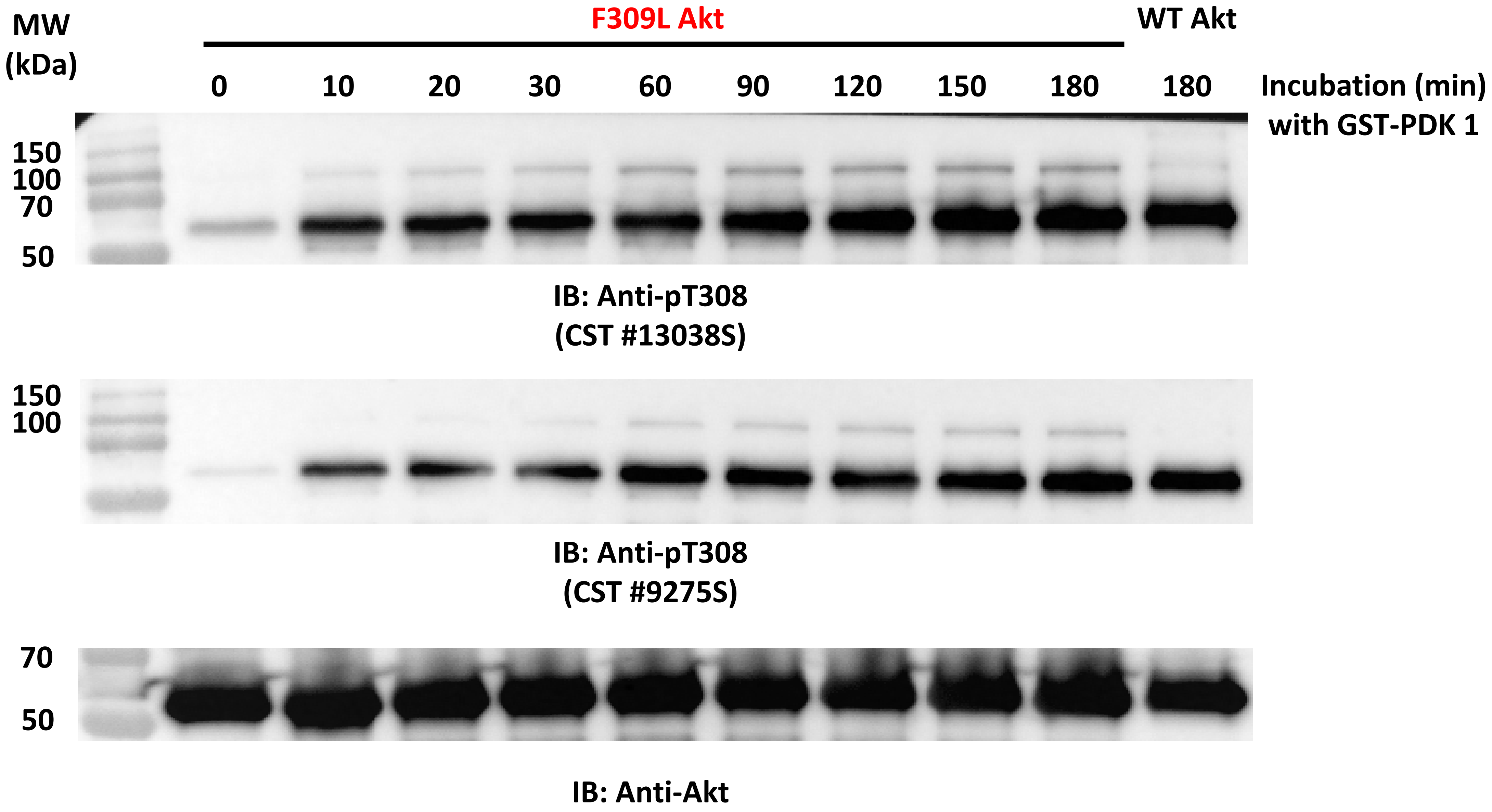

- Western blots were obtained with razor blades to isolate particular regions based on their well-established distinct molecular weights. This is a common and accepted rigorous method done to: 1) ensure that the loading is in fact equal for these experiments in a given lane and 2) to preserve the precious antibody reagents. This is an accepted practice in the field for well-established antibodies like these since they are known to be quite specific. Even if they did light up other bands at different MWs it would not change the conclusions.
