# Supplementary material for: PH domain-mediated autoinhibition and oncogenic activation of Akt
Source: eLife. 2022 Aug 15;11:e80148. doi: 10.7554/eLife.80148 (PMC9417420; doi:10.7554/eLife.80148)
Supplement: Figure 7—source data 1. [file elife-80148-fig7-data1.zip › Figure 7-source data_Cell-based assays (Western blots with labeling).pdf]

Run 1 (biological replicates)

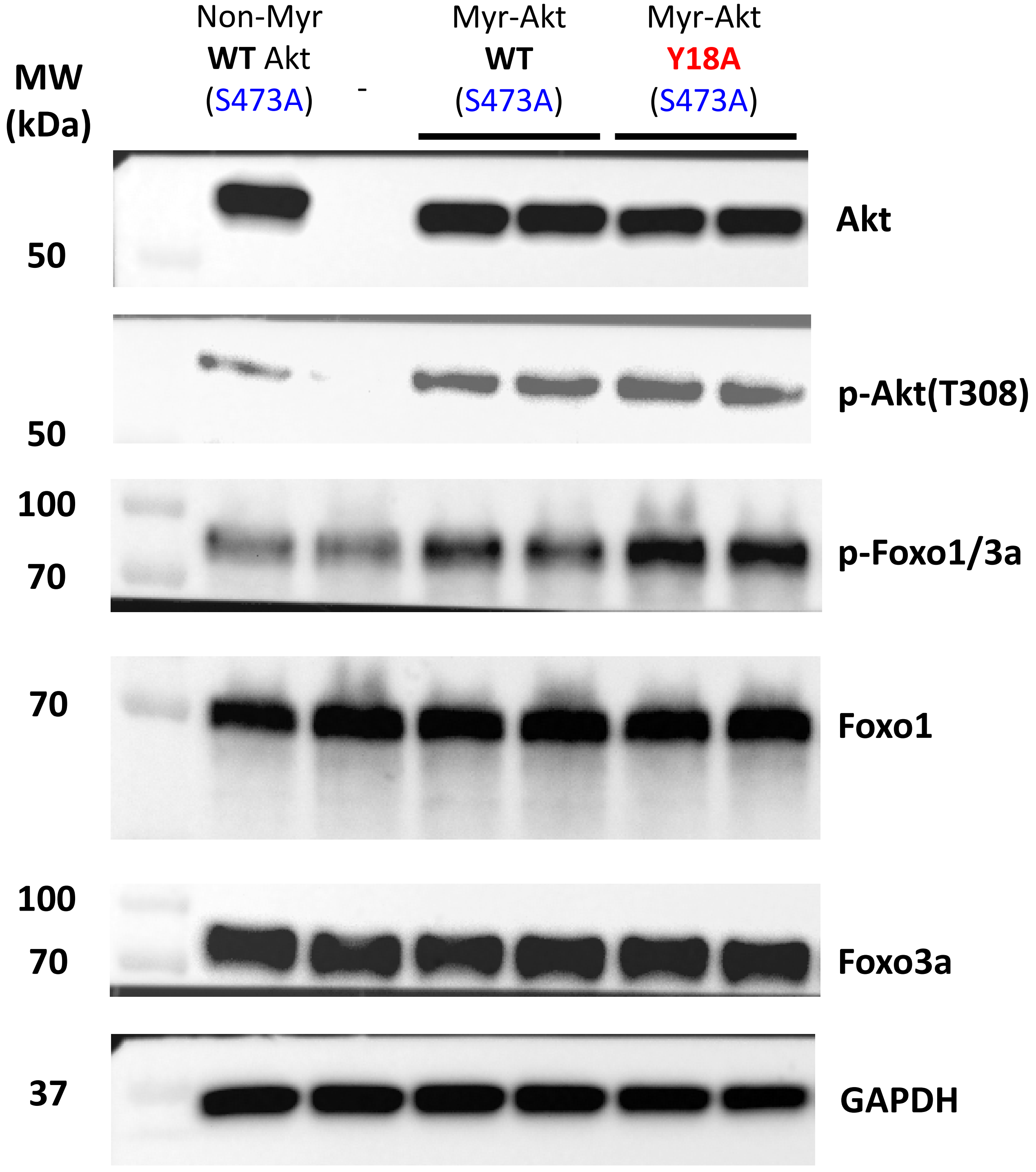

Run 2 (biological replicates)

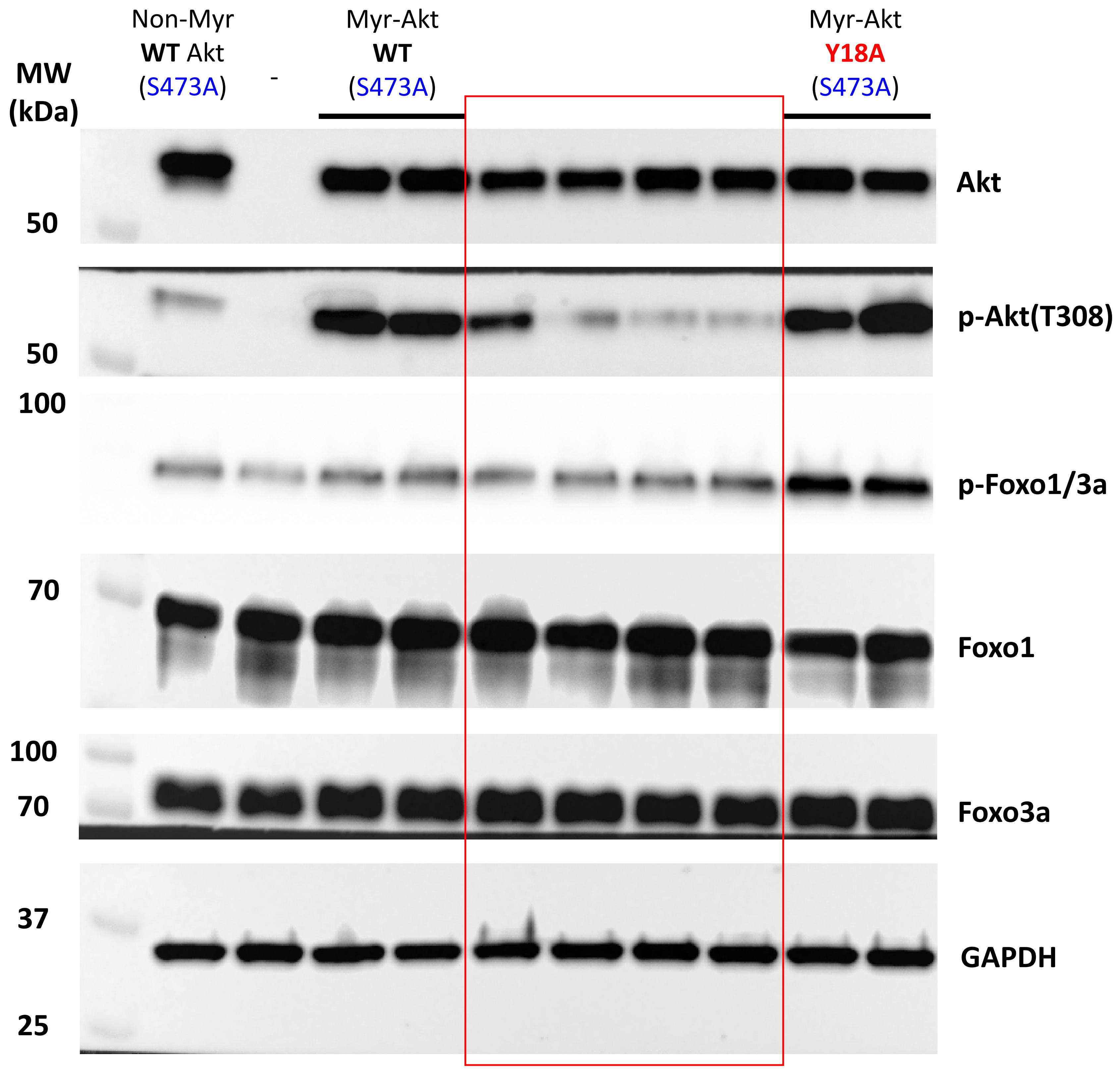

* Western blot result in Red Box was not included in the manuscript

- Western blots were obtained with razor blades to isolate particular regions based on their well-established distinct molecular weights (Akt, Foxo1/3, GAPDH). This is a common and accepted rigorous method done to: 1) ensure that the loading is in fact equal for these experiments in a given lane and 2) to preserve the precious antibody reagents so that multiple gels are not needed for each measurement representing the same cell lysates. This is an accepted practice in the field for well-established antibodies like these since they are known to be quite specific. Even if they did light up other bands at different MWs it would not change the conclusions.
